# Age-dependent bone loss and recovery during hindlimb unloading and subsequent reloading in rats

**DOI:** 10.1186/s12891-018-2156-x

**Published:** 2018-07-18

**Authors:** Hailey C. Cunningham, Daniel W. D. West, Leslie M. Baehr, Franklin D. Tarke, Keith Baar, Sue C. Bodine, Blaine A. Christiansen

**Affiliations:** 1 Biomedical Engineering Graduate Group, University of California Davis, Davis, CA USA; 2 Department of Physiology & Membrane Biology, University of California Davis, Davis, CA USA; 3 Department of Orthopaedic Surgery, University of California Davis Medical Center, Lawrence J. Ellison Musculoskeletal Research Center, 4635 2nd Avenue, Suite 2000, Sacramento, CA 95817 USA; 4 Department of Neurobiology, Physiology, and Behavior, University of California Davis, Davis, CA USA

**Keywords:** Hindlimb unloading, Reloading, Disuse, Age, Trabecular bone, Micro-computed tomography, Mechanical testing

## Abstract

**Background:**

Bone structure and strength are rapidly lost during conditions of decreased mechanical loading, and aged bones have a diminished ability to adapt to increased mechanical loading. This is a concern for older patients that experience periods of limited mobility or bed rest, but the acute effects of disuse on the bones of aged patients have not been thoroughly described. Previous animal studies have primarily examined the effect of mechanical unloading on young animals. Those that have studied aged animals have exclusively focused on bone loss during unloading and not bone recovery during subsequent reloading. In this study, we investigated the effect of decreased mechanical loading and subsequent reloading on bone using a hindlimb unloading model in Adult (9 month old) and Aged (28 month old) male rats.

**Methods:**

Animals from both age groups were subjected to 14 days of hindlimb unloading followed by up to 7 days of reloading. Additional Aged rats were subjected to 7 days of forced treadmill exercise during reloading or a total of 28 days of reloading. Trabecular and cortical bone structure of the femur were quantified using ex vivo micro-computed tomography (μCT), and mechanical properties were quantified with mechanical testing.

**Results:**

We found that Adult rats had substantially decreased trabecular bone volume fraction (BV/TV) following unloading (− 27%) while Aged animals did not exhibit significant bone loss following unloading. However, Aged animals had lower trabecular BV/TV after 3 days of reloading (− 20% compared to baseline), while trabecular BV/TV of Adult rats was not different from baseline values after 3 days of reloading. Trabecular BV/TV of Aged animals remained lower than control animals even with exercise during 7 days of reloading and after 28 days of reloading.

**Conclusions:**

These data suggest that aged bone is less responsive to both increased and decreased mechanical loading, and that acute periods of disuse may leave older subjects with a long-term deficit in trabecular bone mass. These finding indicate the need for therapeutic strategies to improve the skeletal health of elderly patients during periods of disuse.

## Background

Bone undergoes a significant and rapid decay in structure and strength in the absence of mechanical loading [1–3]. In addition, bones of aged humans and animals have a diminished ability to adapt to the mechanical loading environment [4, 5], making exercise or increased mechanical loading less effective at building bone mass. This is particularly important for older subjects that commonly experience periods of limited mobility or skeletal disuse during periods of sickness or following an injury or surgery; the ability of these subjects to regain bone mass following a period of unloading may be compromised.

The effect of mechanical unloading on bone has been extensively studied in both humans and animal models. In humans, a prolonged period of unloading significantly decreases bone volume and bone density as illustrated through bed rest and spaceflight studies [2, 6, 7]. For example, women who participated in 60 day bed rest had decreases of 3–4% in bone mineral density (BMD) in the trabecular bone of the femur and tibia [8]. Similarly, astronauts who spent prolonged time in space experienced a 1–2% trabecular BMD loss in the hip and spine per month in space [9]. Similarly, early models of unloading in mice and rats, including spaceflight studies and tail suspension, found that trabecular and cortical bone volume are rapidly lost during periods of disuse [2, 10–13]. Losses of 20–50% of trabecular bone volume in the proximal tibia have been reported after two weeks of hindlimb unloading in adult rats [11, 14]. In addition, longer bouts of unloading (28 days) have resulted in small (~ 5%) decreases in cortical BMD [13, 15]. While the effect of mechanical unloading on bone has been thoroughly investigated, there have been relatively few studies examining the structural recovery of bone during reloading, and the factors that affect this recovery. Some studies examining reloading indicated that full recovery of BMD requires a reloading period of twice the unloading period [13, 16]. In addition it has been observed in rats that while bone volume returns to control levels during reloading, the bones still have fewer osteoblasts and a lower bone formation rate [11].

The effect of mechanical unloading on aged bone is not well established in either humans or animal models. Human spaceflight studies most often involve healthy young males [6, 7], and there are few bed rest studies using older patients, most of which have investigated the effect on muscle rather than bone [17, 18]. In animal models, few studies have investigated the role of age in bone adaptation to unloading [12], and the effect of age on the reloading response has never been investigated. Most rodent studies have used either young skeletally immature animals (5–7 week old rats) [2, 11, 19] or young adult animals (4–6 month old rats) [13, 15, 20]. It has been suggested that bone of aged rats may not respond at all to short bouts of unloading [12], however this is based on a single study, and there has been no examination of aged animals during longer bouts of unloading or reloading to examine longer-term effects. It is therefore unknown whether aged bone is completely unresponsive to mechanical unloading or if there is a delayed adaptive response to mechanical unloading, and to what extent aged bone is able to recover from any unload-induced bone loss during reloading.

In the current study, we used hindlimb unloading and subsequent reloading of skeletally mature Adult (9 month old) and Aged (28 month old) rats to examine the effect of age on bone loss during mechanical unloading and bone recovery during mechanical reloading. Rats underwent hindlimb unloading for 14 days; this unloading duration has been shown to cause a significant loss of trabecular bone in Adult animals [11, 13], and this unloading period was previously used to examine bone loss in Aged rats [12]. Cortical and trabecular bone microstructure were assessed using micro-computed tomography. Three-point bending and trabecular bone compression were used to assess changes in structural and mechanical properties of cortical and trabecular bone. We hypothesized that Adult rats would exhibit considerable bone loss during unloading, while Aged rats would exhibit less or no significant bone loss as was observed in a past study [12]. We further hypothesized that Adult rats would fully recover bone during reloading, while any bone loss observed following unloading in Aged rats would not be fully recovered during the reloading period. The results of this study could inform interventions such as exercise or mechanical loading for bone fragility in older subjects, particularly in the context of periods of mechanical disuse.

## Methods

### Animals

This study used a total of 63 Fisher 344 x Brown-Norway (FBN-F1) male rats from the National Institute of Aging (NIA) Aged Rodent Colony (Charles River Laboratories, Wilmington, MA). Rats were either 9 months old (“Adult”, *n* = 23), or 28 months old (“Aged”, *n* = 40). All rats were housed individually. Rats were cared for in accordance with the guidelines set by the National Institutes of Health (NIH) on the care and use of laboratory animals. All procedures were approved by the Institutional Animal Care and Use Committee at UC Davis.

### Experimental groups and study design

**Fig. 1:**
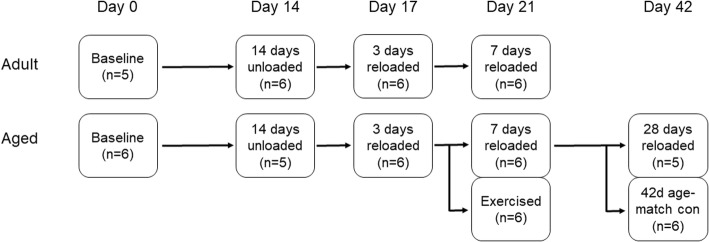
Experimental Design. Adult (9 month old) and Aged (28 month old) rats (*n* = 63) were used for this study. Baseline rats were euthanized at day 0. Remaining groups underwent 14 days of hindlimb unloading, with a group of rats euthanized immediately following hindlimb unloading. All remaining rats were returned to normal cage activity. Rats from both age groups were euthanized after 3 or 7 days of reloading. An additional group of Aged rats were euthanized after 7 days of reloading with timed treadmill walking. Two additional groups of Aged rats were euthanized after 28 days of reloading or at an equivalent age for age-match control (not subjected to hindlimb unloading)

Eleven experimental groups (*n* = 5–6 rats per group) were used to determine the magnitude of bone loss during hindlimb unloading and bone recovery during reloading (Fig. 1). Baseline animals (5 Adult and 6 Aged) were euthanized prior to unloading to determine bone structure before intervention. The remaining rats underwent 14 days of hindlimb unloading via tail suspension. Post-unloading, one group of animals (6 Adult and 5 Aged) was euthanized immediately. Remaining rats were removed from hindlimb suspension and allowed to return to normal cage activity for 3 days (6 Adult and 6 Aged) or 7 days (6 Adult and 6 Aged). Additional groups of Aged rats examined the effect of activity level during reloading, and the effect of longer-term reloading. A group of Aged rats (*n* = 6) underwent 14 days of hindlimb unloading followed by 7 days of reloading with daily treadmill walking (an average of 13.8 m/day) to account for a significant decrease in activity observed in this age group during the first 5 days of reloading. Rats were set to walk on the treadmill at a quick walk (73 m/min) until they had covered the decrease in distance between the rats before and after unloading. Another group of Aged rats (*n* = 5) underwent 14 days of hindlimb unloading followed by 28 days of normal cage activity. To account for age-related decrease in bone, an additional group of Aged rats (*n* = 6) that were not subjected to hindlimb unloading were used as an age-match control to the 28 days reloaded group.

### Hindlimb unloading via tail suspension

Rats were subjected to hindlimb unloading via tail suspension as originally described by Morey-Holten [1]. Briefly, the skin of the tail was cleaned using an alcohol pad, dried, and then sprayed with a light coating of tincture of benzoin. A tail-wide strip of Skin-Trac was then looped around a metal bar and applied to two sides of the tail. The tail was wrapped with Medi-Rip LF self-adherence bandage and then the animal was suspended by attaching the metal bar to a swivel hook above the suspension cage. The height of the hook was adjusted so that only the front limbs were able to touch the bottom of the cage. The rats were able to freely rotate in all directions within their cage and had free access to food and water. Body weights of the rats were taken prior to suspension and monitored throughout the unloading period. Following unloading or reloading, the rats were euthanized and their hindlimbs collected and stored in 70% ethanol prior to analysis. Previous studies have shown that storing bones in ethanol has little effect on the mechanical properties of the bone if properly rehydrated before testing [21, 22].

### Cage activity

Prior to unloading and during reloading, cage activity of Adult and Aged rats was measured using the Home Cage Photobeam Activity System (San Diego Instruments). Each rat was housed individually and activity was tracked throughout the 12-h dark cycle and for the first hour of the light cycle. Data was analyzed using the Photobeam Activity System software.

### Micro-computed tomography analysis of trabecular and cortical bone

Femurs were scanned with micro-computed tomography (μCT 35, Brüttisellen, Switzerland) to analyze morphological changes in trabecular and cortical bone during unloading and reloading. Bones were scanned with x-ray tube potential 55 kVp, intensity 114 mA, integration time 900 ms, 15 μm isotropic nominal voxel size. Trabecular bone analysis was performed at the distal femoral metaphysis over a region of 200 slices (3 mm), starting immediately adjacent to the distal femoral growth plate. Trabecular bone volume fraction (BV/TV), trabecular number (Tb.N), trabecular thickness (Tb.Th), trabecular separation (Tb.Sp), and other structural parameters were calculated using the manufacturer’s 3D analysis software. Cortical bone was analyzed with 200 slice (3 mm) volumes of interest, centered at 25, 50, and 75% of the length of the diaphysis. Bone area (B.Ar), total cross-sectional area (T.Ar), cortical thickness (C.Th), maximum and minimum moments of inertia (I_max_ and I_min_), and other structural parameters were calculated using the manufacturer’s 3D analysis software.

### 3-point bending mechanical testing

Following μCT imaging, femurs were mechanically tested in three-point bending using a materials testing system (ELF 3200, TA Instruments, New Castle, Delaware) to determine cortical bone structural and mechanical properties. Femurs were immersed in phosphate-buffered saline for at least 10 min prior to testing to rehydrate the bone tissue in an isotonic solution. The midpoint of each femur was measured and marked, and each sample was placed in the 3-point bending setup such that the midpoint of the bone was directly under the loading platen. The two lower platens were 13 mm apart, and bones were tested with the posterior aspect of the bone in tension. A single monotonic load to fracture was applied at a slow (quasi-static) loading rate of 1 N/sec. Force and displacement data were recorded at 100 Hz. From the resulting force-displacement data, the yield point was visually identified as the point at which the linear portion of the curve ended. Bending stiffness (K), yield force (F_y_) and ultimate force (F_ult_) were determined from this data. Bone geometry determined from the μCT scan was used to calculate bending modulus (E), yield stress, and ultimate stress.

### Metaphysis trabecular bone compression

After testing with 3-point bending, trabecular bone of distal femurs was mechanically tested in compression as described by Hogan et al. [23]. Distal femurs were potted in bone cement in a small petri dish to hold the bones stationary*.* Approximate location of the growth plate (determined from the μCT images) was marked on the outer surface of the femur. A 3 mm thick slice of the femoral metaphysis was then cut from the region adjacent to the growth plate, including both cortical and trabecular bone. Compression testing was performed with a materials testing machine (ELF 3200, TA Instruments, New Castle, DE). Samples were rehydrated in phosphate-buffered saline for at least 10 min prior to mechanical testing. The bone slice was loaded between two circular platens with diameter of 3 mm such that the platens were only in contact with the trabecular bone and not the cortical bone. Following a preload of 1–2 N, a monotonic (quasi-static) load was applied to the slice at a rate of 0.005 mm/sec. Compression continued to a target displacement of 0.2 mm. Force and displacement data was recorded at a rate of 100 Hz for the duration of the test. From this data, yield and ultimate force, yield and ultimate displacement and stiffness were obtained. Effective stress and effective modulus were obtained by normalizing by the surface area of the platens.

### Statistical analysis

Differences in body weight and cage activity before and after unloading in Adult and Aged animals were analyzed by paired Student’s *t-*test. All other statistical analyses were performed using 2-way analysis of variance (ANOVA) stratified by age and experimental group for main effect, with post-hoc analysis using Tukey’s test. Statistical significance was defined as *p* < 0.05. The additional Aged rat group (7 days reloaded with treadmill walking) was analyzed for significance against the Aged baseline group and Aged 7 days reloaded group with a 1-way ANOVA stratified by experimental group with statistical significance defined as p < 0.05. The additional Aged rat groups of 28 days reloaded and 28 days age-matched control were analyzed for significance against the baseline group with a 1-way ANOVA stratified by experimental group with statistical significance defined as p < 0.05. For the trabecular bone compression test, a linear correlation between trabecular BV/TV and the effective elastic modulus and the correlation coefficient for the correlation was obtained.

## Results

### Animals

Over 14 days of unloading both age groups of rats experienced a significant decrease in body weight. Adult rats lost 12.2% of their body weight on average and Aged rats lost an average of 14.6% of their body weight. Cage activity measurements indicated a significant decrease in distance traveled of an average of 13.8 m/day in the first 5 days of reloading compared to baseline levels in Aged rats [24]; Adult rats exhibited no decrease in activity during reloading.

### Micro-computed tomography analysis of trabecular bone

**Fig. 2:**
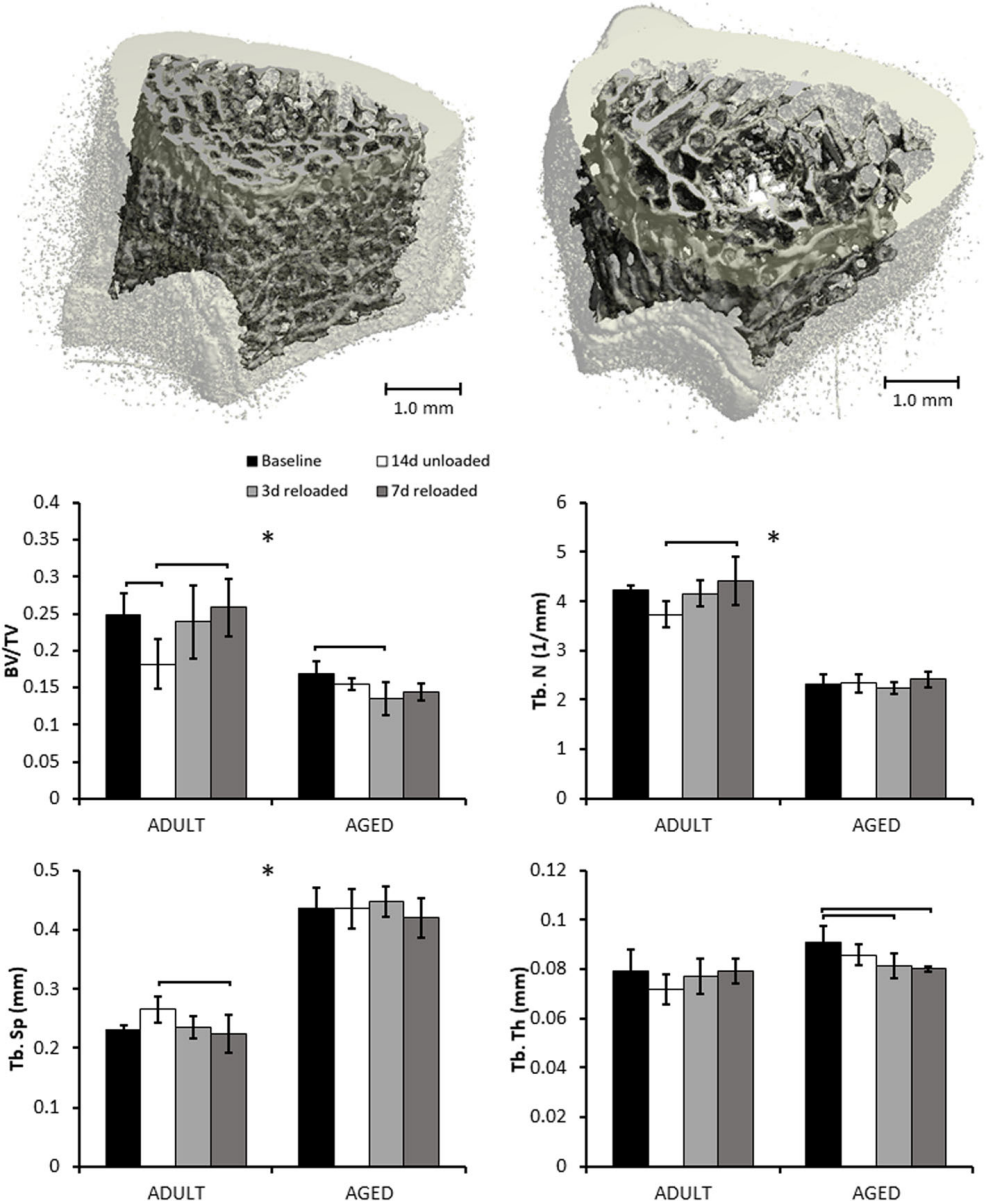
Distal Femoral Metaphysis μCT results. Representative regions of interest of trabecular bone for Adult (left) and Aged (right) femurs at baseline. Adult rats exhibited greater BV/TV, Tb.N, and reduced Tb.Sp than Aged rats. 14 days of hindlimb unloading resulted in significant loss of trabecular bone in Adult rats, but no significant decrease in Aged rats. By day 3 of reloading, Adult rats had recovered trabecular bone volume, while Aged rats exhibited continued loss of bone. Brackets indicate significant differences between groups (*p* < 0.05). Asterisks (*) indicate a main effect of age in a parameter between Adult and Aged animals (*p* < 0.05)

At baseline, bones from Aged rats had significantly less trabecular bone volume than those from Adult rats (0.169 vs. 0.247 BV/TV). Bones from Aged rats also had significantly lower Tb.N and higher Tb.Sp than Adult rats at baseline (Fig. 2). Trabecular thickness at baseline was not different between the two groups. After 14 days of hindlimb unloading, significant differences from baseline were observed in Adult rats but not Aged rats. Bones from Adult rats had 26.6% lower BV/TV (*p* = 0.0493) after 14 days of unloading compared to baseline. In addition, we observed trends toward decreased Tb.N and increased Tb.Sp at this time point, although these were not statistically significant.

After 3 days of reloading, trabecular bone parameters of Adult rats were not different from baseline values. In contrast, Aged bones had 19.7% lower BV/TV than baseline values (0.136 vs. 0.169), and 12.0% lower Tb.Th (*p* = 0.0096) at this time point. After 7 days of reloading, trabecular bone parameters of Adult bones remained similar to baseline values, while trabecular bone parameters from Aged rats remained significantly lower than baseline values.

**Fig. 3:**
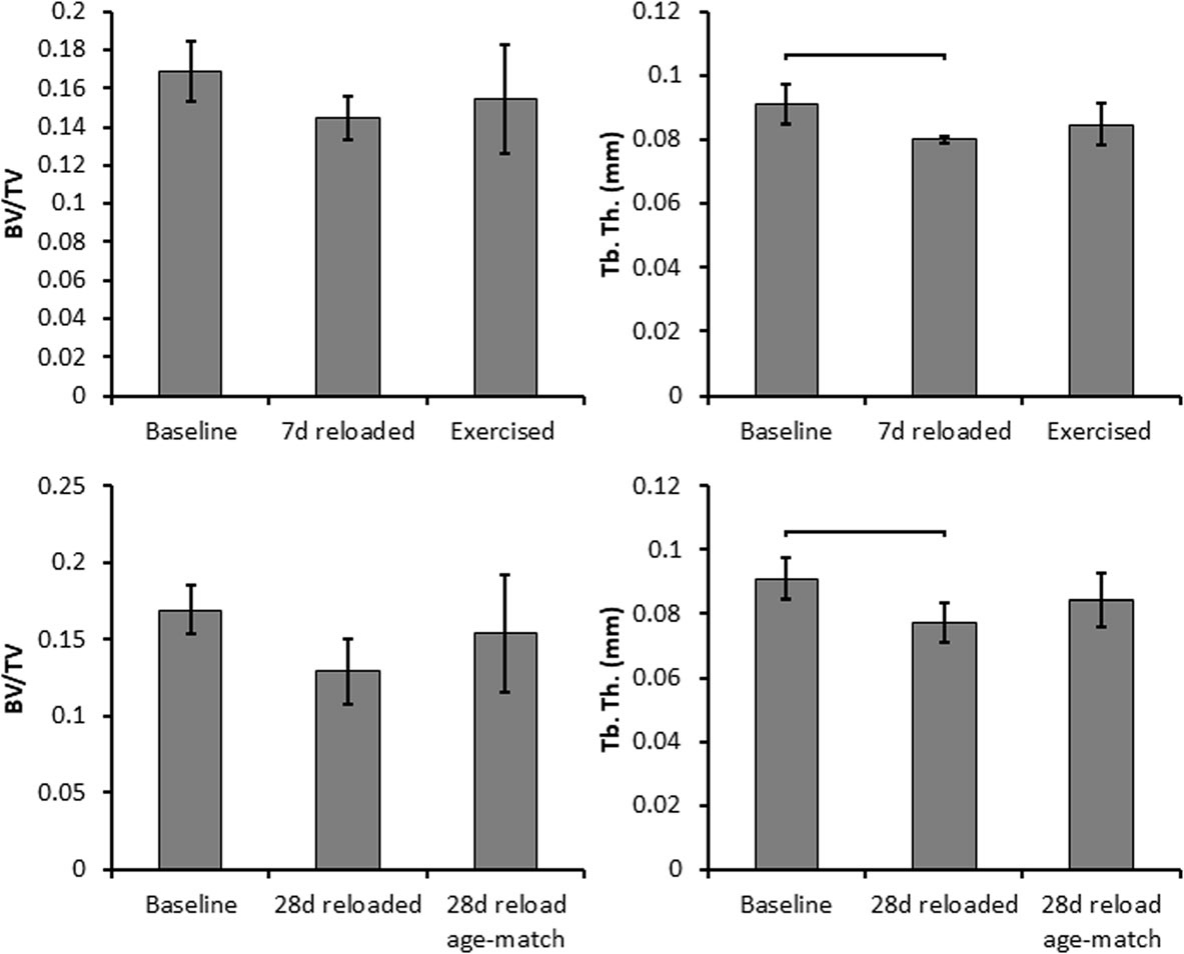
Metaphyseal trabecular bone structure of Exercised and 28 days reloaded Aged rats. 7 days of treadmill walking during reloading did not significantly increase trabecular bone parameters relative to the 7 days reloaded group. Reloading for 28 days did not restore trabecular bone parameters back to control levels. Brackets indicate significant differences between groups (*p* < 0.05)

Aged rats subjected to 14 days of unloading followed by 28 days of reloading, had 15.1% lower Tb.Th than baseline (*p* = 0.0068), and 8.2% lower Tb.Th than the age-matched control group, though this difference was not statistically significant (Fig. 3). Trabecular BV/TV was not significantly different from baseline values after 28 days of reloading.

Seven days of treadmill walking during reloading in Aged rats did not considerably increase trabecular bone parameters compared to the 7 days reloaded group. BV/TV was 6.8% higher and Tb.Th was 5.9% higher in the exercised group than in the 7 days reloaded group, but these increases were not statistically significant.

### Micro-computed tomography analysis of cortical bone

**Fig. 4:**
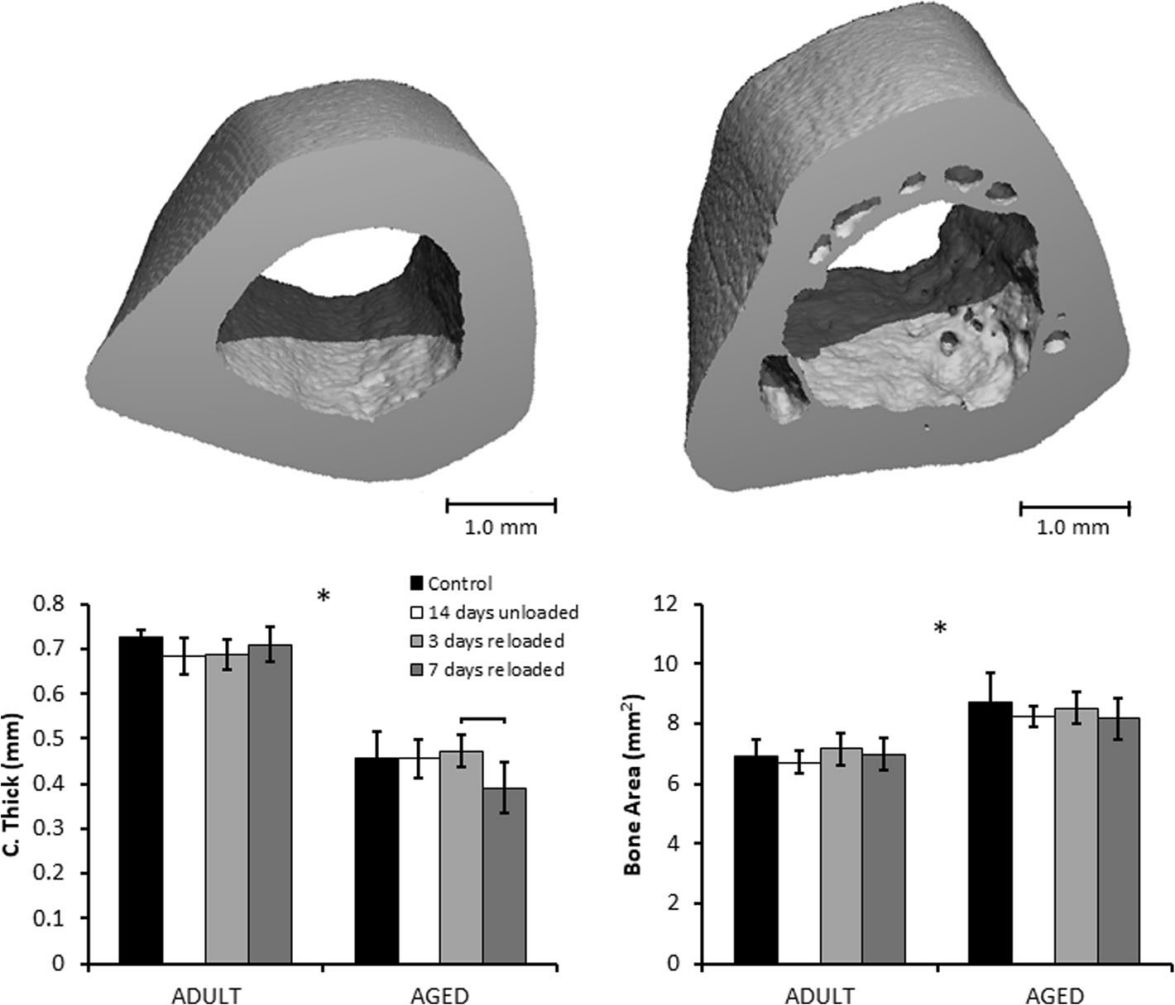
Mid Femoral Diaphysis μCT Results. Representative samples of regions of interest in Adult (left) and Aged (right) femurs. Aged rats had lower average cortical thickness than Adult rats, but greater bone area and total cross-sectional area. Neither age group exhibited changes in cortical structure due to hindlimb unloading or reloading. Brackets indicate significant differences between groups (*p* < 0.05). Asterisks (*) indicate a main effect of age in a parameter between Adult and Aged animals (*p* < 0.05)

Significant differences in cortical bone structure were observed between Adult and Aged rats, however no differences were observed for either age group due to unloading or reloading. Aged rats had significantly lower cortical thickness and significantly higher bone area and total area than Adult rats (Fig. 4). I_max_ and I_min_ were also significantly higher in Aged rats than Adult rats. These observations were consistent across all three regions analyzed.

### 3-point bending mechanical testing

**Fig. 5:**
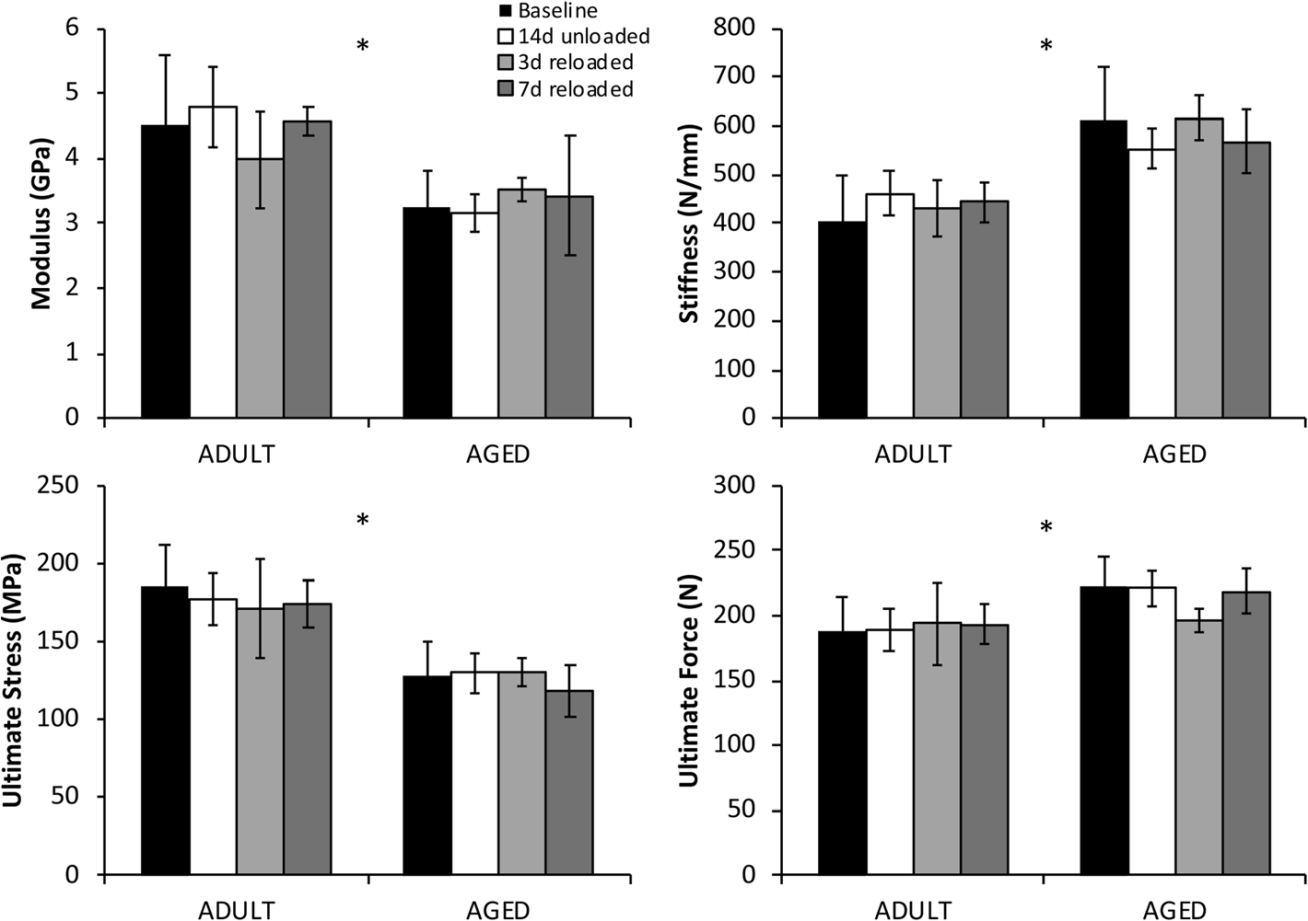
Three-Point Bending Results. Bones from Aged rats exhibited greater stiffness and ultimate force, but lower bending modulus and ultimate stress compared to bones from Adult rats. No significant differences were observed as a result of hindlimb unloading or reloading. Asterisks (*) indicate a main effect of age in a parameter between Adult and Aged animals (*p* < 0.05)

3-point bending yielded similar results to the μCT analysis of cortical bone, with Aged bones generally exhibiting greater structural properties and lower material properties than Adult bones (Fig. 5), but no significant differences in either age group due to hindlimb unloading or reloading. Stiffness and ultimate force were 34.7% higher (*p* < 0.0001) and 11.5% higher (*p* < 0.001) in Aged rats than the Adult rats at baseline, respectively. Yield stress was 39.1% lower (*p* < 0.0001) in Aged rats compared to Adult rats, while ultimate stress similarly was 28.8% lower (*p* < 0.0001) in Aged rats. Elastic modulus was 25.1% lower (*p* < 0.0001) in Aged rats than Adult rats.

### Trabecular bone compression

**Fig. 6:**
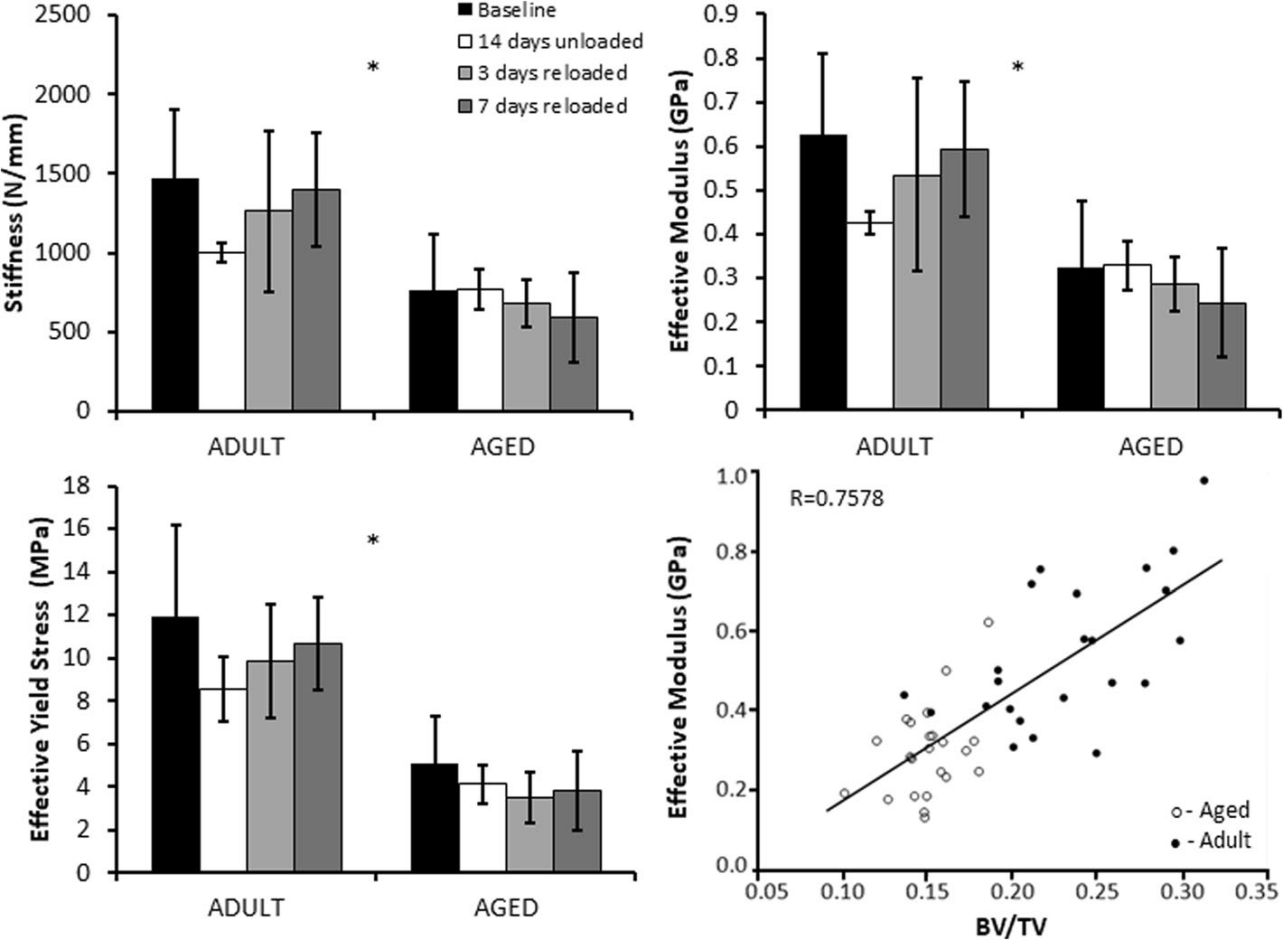
Trabecular Bone Compression Results. Bones from Adult rats exhibited significantly greater stiffness, effective modulus, and effective yield stress than bones from Aged rats. However, there were no significant differences observed as a result of hindlimb unloading or reloading. Effective modulus showed a strong correlation to BV/TV. Asterisks (*) indicate a main effect of age in a parameter between Adult and Aged animals (*p* < 0.05)

Trabecular bone compression yielded trends similar to results of the trabecular bone μCT analysis. However, only age-related differences achieved statistical significance (Fig. 6). Trabecular bone from Adult rats exhibited significantly higher stiffness (+ 82.5%), effective modulus (+ 82.5%), and effective yield stress (+ 146.5%) than those from Aged rats. In the 14 days unloaded Adult rats, we observed trends towards lower stiffness, effective modulus, and effective yield stress compared to baseline values. However, these differences were not statistically significant due to the high variance in the experimental groups. The correlation between BV/TV and effective modulus yielded a correlation coefficient of 0.758, indicating a high correlation between these variables that was statistically significant (*p* < 0.0001) (Fig. 6).

## Discussion

This study investigated difference in bone adaptation to hindlimb unloading and subsequent reloading in Adult and Aged rats. We originally hypothesized that Adult rats would lose more bone compared to Aged animals, and that Aged rats would lose little bone with unloading or even have no apparent bone loss. We also hypothesized that if Aged animals did exhibit bone loss during the unloading period, they would have diminished recovery of bone during reloading. Consistent with this hypothesis, we found that Adult rats showed a marked decrease in trabecular bone volume in response to hindlimb unloading, while Aged rats showed a diminished adaptive response. However, contrary to what has been postulated in previous studies, this does not mean that the Aged rats were necessarily unaffected by unloading. Aged rats did not lose a significant amount of bone volume during the unloading period, but did have lower BV/TV and Tb. Th. than baseline after 3 days and 7 days of reloading. These data suggest that there may be a delay in the adaptation of Aged bone to disuse. Consistent with our initial hypothesis, trabecular bone of Adult rats was not different from baseline levels after 3 days and 7 days of reloading, while bones from Aged rats exhibited deficits in trabecular microstructure even up to 28 days of reloading. These results may suggest that the Aged bones are overall less able to adapt to both unloading and reloading, and that the adaptive response may be temporally delayed. These results may also be a reflection of age-related changes in bone turnover rates and/or bone cellularity, which could lead to different responses to the same periods of unloading and reloading.

The magnitude of trabecular bone loss we observed in Adult rats during hindlimb unloading was similar to previous studies [13, 15]. Decreases in bone formation biomarkers and increases in bone absorption biomarkers have been observed in bed rest studies [25, 26] while in adult rats, bone formation in the hindlimbs has been shown to slow and ultimately cease after one week of tail suspension [27]. In addition, unloading leads to decreased vascularization in the bone, which could impair remodeling and slow recovery during reloading [28]. These are all possible mechanisms for trabecular bone loss observed during unloading in this study, though further studies are needed to confirm these mechanisms.

Few studies have examined the effect of tail suspension on aged animals, and those that have focused solely on unloading with no examination of bone recovery during reloading. One such study found that aged rats are less responsive to unloading and do not lose a significant amount of bone during 14 days of unloading [12]. This is consistent with our observations in this study, in which there was no significant loss of trabecular bone volume during unloading of Aged rats, though a non-significant trend toward bone loss was observed. However, this previous study suggested that aged bone may not respond at all to decreased mechanical stimulation [12]; our current data somewhat contradict these conclusions. While it is true that Aged bones showed no significant decreases in trabecular bone microstructure following the unloading period of our study (14 days), we observed a significant decrease from baseline values in trabecular BV/TV after 3 days of reloading and significant decreases in Tb.Th after 3 and 7 days of reloading. This implies that rather than aged bones being unresponsive to disuse, they may instead experience a delayed reaction to unloading.

It is possible that the gradual loss of trabecular bone in Aged rats could be partially due to normal age-related decreases in bone mass or diminished activity by Aged rats during the reloading period. To address these potential factors, we included a group of Aged rats that were age-matched for the 28 days reloaded group, and a group that was subjected to 7 days of treadmill walking during reloading to restore normal activity levels. We discovered that forcing Aged rats to resume a moderate activity level did appear to lessen some of the bone loss at 7 days of reloading, but did not account for the entire magnitude of bone loss. This implies that a return to a moderate exercise level following periods of disuse could be beneficial in mitigating bone’s delayed reaction to unloading.

In Aged rats there was evidence that some bone recovery eventually occurs during reloading. The 28 days reloaded group had no significant differences in BV/TV from baseline. However, Tb.Th was still lower than baseline after 28 days of reloading in Aged rats. This decrease may be in part due to natural loss of bone due to aging but cannot fully be explained by this since the age-matched control group had trabecular bone parameters that were not significantly different from either the baseline or the 28 days reloading. These data suggest that even after a long reloading period, Aged rats may still have residual deficits in trabecular bone microstructure.

A previous study by Shirazi-Fard et al. showed that over a long period of reloading (28 days or more) in young adult rats, trabecular bone mineral content eventually returns to baseline levels, with recovery requiring twice the unloading time [16]. This is in contrast to what we observed in our study, wherein Adult rats exhibited recovery of trabecular bone volume in a much shorter time frame (3–7 days). It is unclear why these different outcomes were observed. Rats in our study were hindlimb unloaded for 14 days, while the study by Shirazi-Fard used 28 days of hindlimb unloading. This longer period of unloading may have caused biological changes that made it more difficult for trabecular bone to recover during reloading. For example, it has been observed that while bone volume may return to control levels, reloaded bones still have fewer osteoblasts and a lower bone formation rate than control bones [11], indicating that there are important long-term biological effects of unloading. Our current study did not examine changes in cellular activity in these bones, therefore we cannot determine if osteoblast number or activity were altered. Importantly, our study used a cross-sectional study design, so longitudinal changes are inferred using different groups of rats, rather than tracking the same group of rats over time. In contrast, the study by Shirazi-Fard [16] used both longitudinal assessment of bone structure and cross-sectional bone imaging and mechanical testing. It is difficult to say how accurately the inferred longitudinal changes in our study represent the true magnitudes and timeline of adaptive bone changes.

In this study, we observed that cortical and trabecular bone adapted very differently to changes in the mechanical loading environment. Differences in trabecular bone microstructure were easily detected in Adult rats immediately following unloading and at early time points during reloading, while Aged rats exhibited differences in trabecular microstructure at later time points. In contrast, cortical bone did not exhibit any adaptation to changes in loading at any time points. This is consistent with previous studies that have found that hindlimb unloading in rats and mice has an effect on cortical bone only for unloading bouts of longer than 14 days [15]. Considering the lack of cortical bone adaptation determined by μCT, it was not surprising that three-point bending showed no changes in mechanical properties as a function of unloading and reloading. In trabecular bone mechanical testing, we anticipated a similar trend to the changes in trabecular bone structural parameters from the μCT analysis. The high variances in the test resulted in no significant differences between experimental groups, however we were able to demonstrate that the mechanical properties of the trabecular bone were highly correlated to the structural properties found in μCT analysis. In this study we observed significant age-related differences in structural and material properties between Adult and Aged rats. The structural differences we observed, such as lower Ct.Th and larger I_min_, and I_max_ in Aged rat bones, are consistent with previous studies in which bones from older animals had larger diameters but thinner cortical shells [29, 30]. We also observed that stiffness was higher in Aged animals while modulus and ultimate stress were lower; these data are also consistent with previous studies [29, 31].

The findings of this study have significant implications for aged patients. During sickness, or following an injury or surgery, older patients may spend a prolonged period in bed rest or with limited movement. Such a period of disuse could result in a loss of bone mass, which has previously been confirmed in human studies [8, 25]. Our data suggests that there may be a delay in the skeletal response to a decrease in mechanical loading, and bone loss may continue even during reloading. Clinically, this delay would be concerning as elderly patients who are remobilizing after bed rest may be more vulnerable to subsequent fracture. Our findings may motivate a rethinking of how elderly patients are treated during bed rest or limited mobility, and may imply that simply a return to normal activity levels following a period of disuse may not be enough to fully regain skeletal health.

While this study makes strong conclusions about differences between aged and adult bone in response to unloading and reloading, there are a number of limitations that must be addressed. First, this was a cross-sectional study, and did not examine the same animals at baseline, following unloading, and during reloading. Therefore, longitudinal changes due to unloading and reloading must be inferred based on cross-sectional data from different experimental groups. Second, we did not directly analyze bone formation or bone resorption rates, or other measures of cellular activity in these rats. Even with this limitation, we were able to determine significant differences in trabecular bone as a function of unloading and reloading, and differences in both cortical bone and trabecular bone as a function of age. Third, the group sizes (5–6 animals per group) were small. As a result of the small group sizes, several biologically meaningful measures did not yield statistically significant results. However, even with limited sample size, we were able to demonstrate differences between how aged and adult bones respond during the unloading-reloading timeline. Finally, this study did not examine other factors that could contribute to bone remodeling during unloading and reloading such as changes in food and water consumption, changes in gait, or effects of muscle atrophy on loads applied to the bones by muscles.

## Conclusions

This study is one of few studies to examine the effect of age on bone adaptation to mechanical unloading, and the first to examine bone adaptation during subsequent reloading as a function of age. We demonstrate that bones from Aged animals have a delayed and diminished adaptive response to mechanical unloading, and an impaired capacity to recover bone during reloading. The delayed response of Aged bone could have meaningful consequences with respect to fracture risk and skeletal health of elderly patients, and should be considered for therapies aimed at preserving bone health during periods of disuse and subsequent remobilization.

## Abbreviations

B.Ar: Bone area
BMD: Bone mineral density
BV/TV: Trabecular bone volume fraction
C.Th: Cortical thickness
F_ult_: Ultimate force
F_y_: Yield force
I_max_: Maximum moment of inertia
I_min_: Minimum moment of inertia
T.Ar: Total cross-sectional area
Tb.N: Trabecular number
Tb.Sp: Trabecular separation
Tb.Th: Trabecular thickness
μCT: Micro-computed tomography
